# Transition to Multidisciplinary Pediatric Telerehabilitation during the COVID-19 Pandemic: Strategy Development and Implementation

**DOI:** 10.3390/ijerph18041484

**Published:** 2021-02-04

**Authors:** Tal Krasovsky, Tamar Silberg, Sharon Barak, Etzyona Eisenstein, Neta Erez, Irit Feldman, Dafna Guttman, Pnina Liber, Smadar Zohar Patael, Hadar Sarna, Yaara Sadeh, Pnina Steinberg, Jana Landa

**Affiliations:** 1 Department of Physical Therapy, University of Haifa, Haifa 3498838, Israel; 2 Department of Pediatric Rehabilitation, the Edmond and Lily Safra Children’s Hospital, Sheba Medical Center, Ramat-Gan 5262000, Israel; tamarsilberg@gmail.com (T.S.); sharoni.baraki@gmail.com (S.B.); Etzyona.Eisenstein@sheba.health.gov.il (E.E.); netaere@gmail.com (N.E.); iritfeldman5@gmail.com (I.F.); dafna.gutman@sheba.health.gov.il (D.G.); pninaliber@gmail.com (P.L.); smadar.pa@gmail.com (S.Z.P.); Hadar.simana@sheba.health.gov.il (H.S.); yaarakraus@gmail.com (Y.S.); pninasteinberg@gmail.com (P.S.); Janna.Landa@sheba.health.gov.il (J.L.); 3 Department of Psychology, Bar-Ilan University, Ramat-Gan 52900, Israel; 4 Kaye Academic College of Education, M.Ed Programs and Physical Education Program, Beer-Sheva 8414201, Israel; 5 Department of Communication Disorders, Steyer School of Health Professions, Sackler Faculty of Medicine, Tel Aviv University, Tel Aviv 6997801, Israel; 6 The Louis and Gabi Weisfeld School of Social Work, Bar-Ilan University, Ramat-Gan 52900, Israel; 7 Sackler Faculty of Medicine, Tel Aviv University, Tel Aviv 6997801, Israel

**Keywords:** telehealth, children, physical therapy, occupational therapy, COVID-19, coronavirus, presence

## Abstract

Telerehabilitation offers a unique solution for continuity of care in pediatric rehabilitation under physical distancing. The major aims of this study were to: (1) describe the development of telerehabilitation usage guidelines in a large hospital in Israel, and to (2) evaluate the implementation of telerehabilitation from the perspectives of healthcare practitioners and families. An expert focus group developed guidelines which were disseminated to multidisciplinary clinicians. Following sessions, clinicians filled The Clinician Evaluation of Telerehabilitation Service (CETS), a custom-built feedback questionnaire on telerehabilitation, and parents completed the client version of the Therapist Presence Inventory (TPI-C) and were asked to rate the effectiveness of sessions on an ordinal scale. Four goals of telerehabilitation sessions were defined: (1) maintenance of therapeutic alliance, (2) provision of parental coping strategies, (3) assistance in maintaining routine, and (4) preventing functional deterioration. Principal Components Analysis was used for the CETS questionnaire and the relationships of CETS and TPI-C with child’s age and the type of session were evaluated using Spearman’s correlations and the Kruskal–Wallis H test. In total, sixty-seven telerehabilitation sessions, with clients aged 11.31 ± 4.8 years, were documented by clinicians. Three components (child, session, parent) explained 71.3% of the variance in CETS. According to therapists, their ability to maintain the therapeutic alliance was generally higher than their ability to achieve other predefined goals (*p* < 0.01). With younger children, the ability to provide feedback to the child, grade treatment difficulty and provide coping strategies to the parents were diminished. Families perceived the therapist as being highly present in therapy regardless of treatment type. These results demonstrate a potential framework for the dissemination of telerehabilitation services in pediatric rehabilitation.

## 1. Introduction

Following the rapid global spread of the new acute respiratory syndrome coronavirus 2 (SARS-CoV-2), the virus that causes novel coronavirus disease 2019 (COVID-19), health care systems were required to endorse prompt transitions, both in the internal structure and in the strategy and approach of their care delivery [1]. Subsequently, many facilities found themselves rapidly expanding telehealth infrastructure and applications [2]. Telehealth is defined by the US Department of Health and Human Services as “the use of electronic information and telecommunication technologies to support long-distance clinical health care, patient and professional health-related education, public health and health administration” [3]. This broad definition encompasses a wide variety of media such as text messages, telephone or videoconferencing, and can be delivered either in a synchronous (real-time) or asynchronous (store-and-forward) manner [4]. Telehealth services have been increasingly used when dealing directly with COVID-19 patients as well as with other patients within the health system. Specifically, due to physical distancing measures administered during the spread of the pandemic, children and adolescents who required rehabilitation services were encouraged to stay at home rather than remain hospitalized or visit a hospital environment that may endanger them. These patients can be those who underwent serious injuries, such as brain injuries, orthopedic injuries or burns, which would have necessitated prolonged inpatient rehabilitation, or children presenting with chronic conditions such as cerebral palsy and requiring ongoing rehabilitation care. In both cases, telerehabilitation, defined as the provision of rehabilitation services using telehealth, offers a unique solution which can provide continuity of care [5].

Among people with various health conditions, the provision of services via telehealth is generally considered at least as effective as face-to-face therapy. This is the case for people with stroke [6,7], people with heart conditions [8] and musculoskeletal conditions [9], or after surgery [10,11]. The advantages of telehealth are numerous, including an ability to overcome geographical barriers and reduce costs of therapy. However, despite the advantages and successes of telehealth, its provision to date is not as widespread as could be expected [12], and it is estimated, for example, to be utilized in less than 1% of specialist consultations in Australia [13]. It is assumed that the low uptake of telehealth services stems from problematic business (reimbursement) models and low levels of acceptance from clinicians [14,15]. Low acceptance levels of clinicians may be due to the fact that the use of telehealth requires new competencies from clinical staff, such as “knowledge about what to do if the technology does not work”, or “to communicate clearly in videoconferencing” [16]. The requirement to learn new skills may cause anxiety in health professionals and prevent the efficient uptake of technology. Furthermore, unique challenges for deployment of telehealth services emerge when dealing with specific populations. For example, among some pediatric populations, it has been reported that children and adolescents with Autism Spectrum Disorder demonstrate satisfaction with telehealth solutions and their effectiveness in terms of behavior [17,18,19]. However, the literature on implementation and dissemination of telehealth in children with physical and cognitive impairments who require rehabilitation services has, to date, been sparse [20].

Considering the ability of telerehabilitation to maximize rehabilitation potential, and the scarcity of studies in the literature on telerehabilitation in the pediatric population, further insights on this topic are necessary [20]. Moreover, the deployment of telerehabilitation services, especially under extreme conditions, needs to follow specific guidelines in order to achieve treatment goals. In situations of uncertainty and stress among families and healthcare workers, this may be a complex task. Thus, the objectives of the current work were to: (1) describe the development of telerehabilitation usage guidelines in a large pediatric hospital in Israel, and to (2) evaluate the implementation and usage of telerehabilitation from the perspectives of healthcare practitioners and families. Understanding the unique experience of staff and families during the initial period of transition to telerehabilitation can assist in developing best-practice models of pediatric telerehabilitation for days after the pandemic.

## 2. Materials and Methods

Israel began enforcing social distancing and other actions to limit the spread of the virus beginning on 11 March 2020. On 17 March, partial lockdown was announced, and the first COVID-19-related death was reported on March 20. From 25 March until 19 April, a full lockdown was initiated, barring citizens from walking >100 m from home unless for urgent needs. During that time, the number of COVID-19-related cases in Israel grew from 2465 (# of deaths = 5) to 13,884 (# of deaths = 171). This work was initiated in March and comprised two stages: (1) development of guidelines, and (2) evaluation of telerehabilitation sessions which were carried out until the end of April. Since, in early May, face-to-face visits were largely possible, telerehabilitation services were then diminished significantly. The current study is part of a larger study aimed at evaluating risk and protective factors associated with the adjustment of the Pediatric Rehabilitation Department to the COVID-19 pandemic. 

### 2.1. Development of Guidelines for Telerehabilitation Practice

First, a focus group was organized based on diverse staff members and heads of clinical sectors at the department, comprising N = 15 members: physicians (including department head), researchers, head of psychology, head of physiotherapy, head of occupational therapy, head of speech therapy, head of nursing, head of social workers, an educator and a medical anthropologist. Selection of focus group members was based on level of clinical experience (consisting of heads of all clinical sectors within the department) and diversity, intended to incorporate different areas of clinical and research expertise. This focus group met (via online communication) once or twice weekly to define and promote telerehabilitation usage guidelines. Discussions were structured around a set of carefully predetermined questions and moderated by one of the researchers, skillful in facilitating group discussions. For example, some questions discussed were “what are the principles guiding usage of telerehabilitation under the current conditions”, “what would be the optimal duration of time and the relative division of time for different components of rehabilitation (e.g., parental guidance, assessment of functioning of the child, etc.) within the therapeutic session”, “what specific guidelines should be given to parents within and outside of therapy sessions (e.g., availability of the parent during treatments, between-sessions guidelines for working with the child)”, “what criteria need to be met by families in order to facilitate telerehabilitation (e.g., characteristics of child and family)”.

Second, due to the need to produce information in a timely manner, a rapid review of the literature was conducted in order to assess existing literature on use of telerehabilitation in pediatrics and to facilitate the focus group discussions. Databases searched included electronic databases, such as MEDLINE, EMBASE, and Cochrane Central Register of Controlled Trials. Studies were included if they were published between 2015 and April 2020 and written in English. Quantitative, qualitative, case reports, and mixed-method studies were included in order to consider different aspects of telerehabilitation within the pediatric population. Literature review keywords used were: telehealth, telerehabilitation, pediatric, healthcare, children, remote rehabilitation. Based on results of the rapid review and clinical experience, the focus group arrived at a consensus document which detailed goals for telerehabilitation and guidelines for implementation. The highlights from the document are provided in Table 1. The document was administered via leaders of clinical sectors to clinicians, and adaptations were made in order to include strategies, specific to each sector, in order to reach goals.

### 2.2. Evaluation of Telerehabilitation Sessions

**Staff**: A short feedback questionnaire was developed by members of the focus group in order to assess telerehabilitation sessions. The Clinician Evaluation of Telerehabilitation Service (CETS) questionnaire comprised 13 questions, which were agreed upon by members of the focus group and related broadly to the content of the guidelines provided to staff (Table 1). Specifically, questions were related to the ability to attain the prespecified therapeutic goals, the content of the session, ability to assess the function of the child and the affordances (environmental, personal) of the therapeutic session participants. Each question was rated on a 5-point Likert scale ranging from “not at all” to “A lot”. Questionnaire development was based on the available literature and input from the focus group and the clinicians. Corrections to the first draft of the questionnaire were based on feedback from the focus group until consensus was reached between entire focus group members.

Clinicians (physical, occupational therapists and speech language pathologists) were asked to fill the CETS following telerehabilitation sessions, and responses were subsequently also used as a clinical decision-making aid; clinicians were encouraged to collaborate and learn from the experiences of others in telerehabilitation by providing access to responses of the CETS. To this end, responses were not anonymized and were available to clinicians of all sectors. In addition to the structured questionnaire, clinicians were able to report, in free text, ideas they had for sessions and specific barriers they identified, in the hope of providing useful information for clinicians in different sectors treating the same child.

**Families**: In an attempt to receive families’ feedback with telerehabilitation, while reducing the burden on family-participants as much as possible, a short survey was administered. Parents/caregivers of children participating in telerehabilitation were requested to complete the Hebrew version of the Therapist Presence Inventory-Client version (TPI-C). TPI-C is a 3-item scale used to assess clients’ experience of therapist presence during the last session using a 7-point Likert scale ranging from 1—“not at all “ to 7— “completely” [21]. In addition, families completed a single question relating to how effective the telerehabilitation session was on a scale from 1—“not at all” to 7 “very effective”. Families were also able to add, in free text, any comments they had regarding their personal experience with telerehabilitation.

### 2.3. Data Analysis

Responses to the questionnaires were investigated using descriptive statistics. Principal Components Analysis (PCA) was used for the CETS questionnaire, in order to identify common themes associated with usage of telerehabilitation services among staff. An orthogonal (varimax) rotation was used to maximize variance in order to identify themes. Before conducting the PCA, various statistical assumptions necessary for PCA were tested [22]. The Kaiser–Meyer–Olkin (KMO) index of sampling adequacy was set at >0.75. Bartlett’s test of sphericity has to be highly significant (*p* < 0.001) [23]. The optimal number of factors was determined by latent root criteria (eigenvalues > 1.0, the Kaiser’s criterion K1) and inspection of the scree plot [22]. Scores of factors identified within the questionnaire, as well as responses to specific questions (regarding goals), were compared using Friedman’s tests and Wilcoxon ranked sum tests. In addition, the relationship of the age of the child and the type of session with CETS responses was evaluated using Spearman’s correlations and the Kruskal–Wallis H test. Scores on the TPI-C (family’s feedback) were examined descriptively and the relationships of TPI-C with the child’s age and the type of session (e.g., physical, occupational, speech therapy) were examined using Spearman’s correlations.

## 3. Results

### 3.1. Development of Guidelines for Telerehabilitation Practice

**Figure 1:**
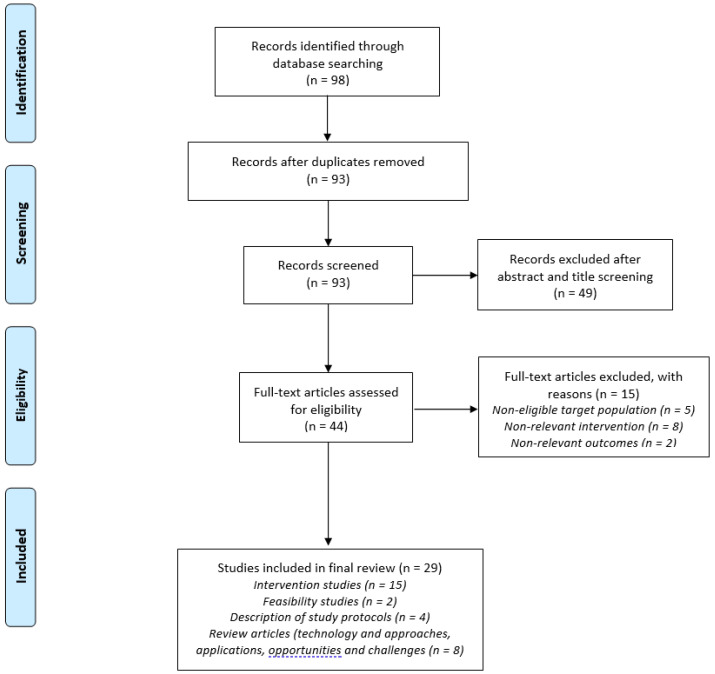
Flow chart according to the Preferred Reporting Items for Systematic Reviews and Meta-Analyses (PRISMA) method for the literature review search process which was conducted. See text for details [24].

Ninety-eight reports were retrieved from the literature review. The 98 reports yielded 29 eligible articles. Reports’ exclusion was mainly attributed to non-relevant interventions (e.g., home-based computerized training, supervised outpatient programs, and virtual reality interventions), non-eligible target population (e.g., adults), and duplicate reports. The included reports mainly consisted of telerehabilitation-related intervention studies in various pediatric populations (e.g., children with chronic diseases and physical disabilities) and review articles on pediatric telerehabilitation (e.g., technology and approaches, applications, and opportunities and challenges; Figure 1). From the literature review it was clear that telemedicine and telehealth solutions are emerging rapidly in health care; however, the identified publications used different kinds of technological solutions, addressed different populations (e.g., cystic fibrosis, cardiac conditions, obesity, etc.) with limited reference to moderate–severe physical limitations, and there was no agreement on guidelines for the use of telerehabilitation in pediatrics. Finally, none of the identified literature addressed telerehabilitation usage under extreme conditions of lockdown due to a pandemic.

Following the work of the focus group and based on the literature review, four general goals for the provision of telerehabilitation were defined across all sectors: (1) maintenance of the therapeutic alliance, (2) provision of coping strategies for parents, (3) assistance in maintaining routine, and (4) prescribing measures to prevent functional deterioration. These goals, and the principles of telerehabilitation treatment defined by the focus group (Table 1), were then disseminated to staff by heads of the different sectors. A total of N = 50 families were offered telerehabilitation based on medical decisions involving the families’ ability to engage in telerehabilitation (e.g., fast internet connection, approach to technology), and the child’s cognitive and behavioral state. Out of the 50 families, 35 were still receiving telerehabilitation on 30th April. Fifteen families opted out of telerehabilitation due to bureaucracy (N = 5), religious background (N = 5), age (N = 2) or lack of effectiveness (N = 3).

**Figure 2:**
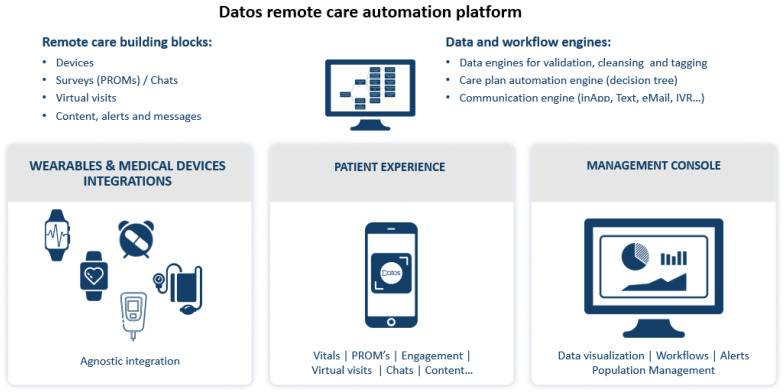
Description of Datos remote care automation platform used in the current study. In the pediatric rehabilitation department during the pandemic, and in order to facilitate connectivity, only the virtual video call functionality was used (no wearables/other medical devices were integrated).

A total of N = 140 telerehabilitation sessions took place at the Pediatric Rehabilitation Department at Sheba Medical Center between 25th March and 30th April, out of which N = 67 were documented by clinicians for the study using CETS. All telerehabilitation sessions complied with federal and hospital regulations for the protection of client health information and to ensure the security of electronic data storage, retrieval, and transmission. Specifically, sessions were conducted using the Datos remote care platform (Datos Health, Tel-Aviv, Israel) as per the hospital’s regulations. Datos is a fully U.S. Food and Drug Administration (FDA) and Health Insurance Portability and Accountability Act (HIPAA) compliant automated remote care platform, which enables hospitals to deploy remote care processes and workflows. Datos’ agnostic platform enables care delivery across different clinical conditions, devices and protocols and creates strong patient engagement (Figure 2; www.datos-health.com). In the current study, Datos’s virtual video call functionality was used to provide telerehabilitation sessions to families in the home setting. Following the sessions, thirty-six parents/caretakers completed the TPI-C questionnaire as well as evaluating the effectiveness of telerehabilitation using the single-item question.

### 3.2. Evaluation of Telerehabilitation Sessions—Clinicians and Families

Clinicians reported on 67 telerehabilitation sessions conducted between 24th March and 30th April 2020. Thirty-nine were physiotherapy sessions, 13 were occupational therapy sessions, 6 were remote learning sessions, 5 were speech therapy sessions and 4 were medical consultations. Most sessions (29.9%) lasted 30–40 min, 23.9% lasted under 30 min and 7.5% lasted 45–60 min. In 88.1% of cases, the child was previously seen in person for evaluation and/therapy (pre-COVID-19). Participating children’s average age was M = 11.31 ± 4.81 (range 0.2–18 years). The Cronbach’s alpha for the total score of the feedback questionnaire was good (α = 0.712).

To evaluate factors associated with the variance in responses to the CETS questionnaire, a PCA was conducted. The suitability of PCA was assessed prior to analysis. The correlation matrix showed that all variables had at least one correlation coefficient greater than 0.3. The overall Kaiser–Meyer–Olkin (KMO) measure was 0.703 and Bartlett’s Test of Sphericity was statistically significant (*p* < 0.0005), indicating that the data were likely factorizable.

PCA revealed three components that had eigenvalues greater than one and which explained 45.2%, 17.67% and 8.41% of the total variance, respectively, for a total of 71.3% of variance explained (Table 2). A Varimax orthogonal rotation was used, resulting in an interpretation of the data which was consistent with the aspects the questionnaire aimed to measure. Specifically, items related to the child were loaded on Component 1, those related to the session itself were loaded on component 2, and those related to the parent’s involvement were loaded on Component 3. Scores on the three components of the scale varied significantly ($\chi_{2}\left(3\right)=24.3,\;p<0.001$) such that the Parent (component 3) scores were higher than Session (component 2; Z = −3.5, *p* < 0.001) and Child (Component 1; Z = −4.11, *p* < 0.001) and the Session was higher than the Child as well (Z = −3.0, *p* < 0.01), with no differences between sectors. The child’s age was positively associated with their cooperation (r = 0.47, *p* < 0.01), the effectiveness of feedback (r = 0.48, *p* < 0.01) and the ability to grade levels of difficulty (r = 0.37, *p* < 0.01) and negatively associated with the level of child’s distraction (r = −0.46, *p* < 0.001) and with the ability of the session to provide coping strategies for the parent (r = −0.35, *p* < 0.05).

**Figure 3:**
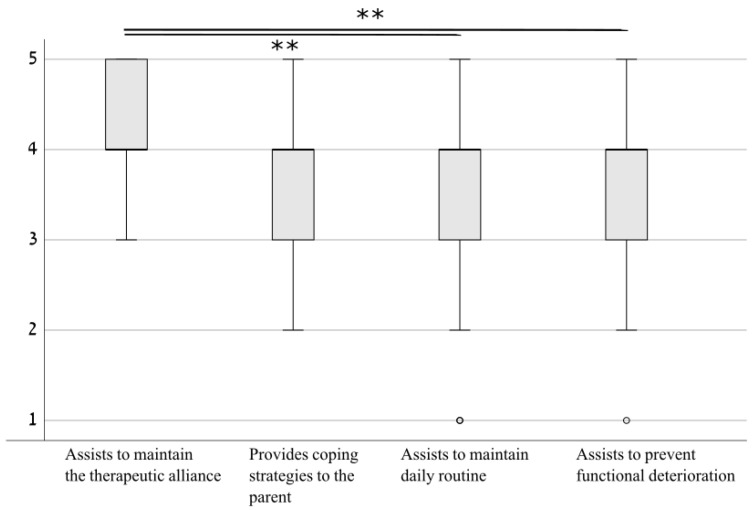
Adherence to pre-defined goals for telerehabilitation. Results demonstrated that the ability to maintain the therapeutic alliance was superior to the ability to assist in maintaining routine and to prevent functional deterioration, and marginally superior to the ability to provide coping strategies to the parent. ** *p* < 0.01.

A comparison of responses to the four questions relating to the stated goals of telerehabilitation (Figure 3) demonstrated that the degree to which telerehabilitation was able to meet these goals differed by goal ($\chi_{2}\left(3\right)=12.7,p=0.005$) and post-hoc tests demonstrated that the ability of telehealth to maintain the therapeutic alliance was significantly larger than the ability to attend to two of the other goals (assistance in maintaining routine: Z = −3.4, *p* = 0.001; assistance in reducing functional deterioration: Z = −4.0, *p* < 0.001) and marginally higher than the ability to provide coping strategies for parents (Z = −1.8, *p* = 0.065). Division by sector emphasized that this difference was identified in both physical and occupational therapy sessions (Z = −2.3 to Z = −2.7, *p* = 0.022) but not in medical consultations, educational or speech therapy, where all goals were similarly attained.

When asked to detail barriers to telerehabilitation sessions, many clinicians attested to increased distractibility of the children. In some cases, this distractibility was not specific to telehealth (i.e., the child is known to have had attentional or cognitive issues prior to COVID-19). In some cases, distractions were described as associated with the home environment (e.g., siblings, other sources of noise) or technology (e.g., difficulty to listen to the therapist due to communication problems or increased movement of the child). Clinicians reported difficulty in performing a formal functional assessment, lowering their ability to determine the child’s functional status, which is an important factor in rehabilitation settings. In some cases, environmental factors such as a small room or lack of equipment were described as limiting the ability to practice and understand the quality of performance of motor tasks. In contrast, clinicians detailed previous acquaintance with the child in face-to-face sessions as a facilitator of treatment effectiveness. Some environmental factors (e.g., siblings’ engagement or physical characteristics of the home, e.g., the child’s personal games) were also described as facilitators of treatment.

The Cronbach’s alpha for the TPI-C in the present study was moderate (0.547). Parents reported that they had perceived the therapist’s presence in the telehealth sessions as extremely high on all items of the TPI-C (Table 3), with no significant differences between the type of therapy delivered (U = 88.05, *p* = 0.31). In 75% of cases, the scores provided were the highest possible for all questions. When asked about the effectiveness of telerehabilitation settings, parents reported that they perceived the telerehabilitation sessions as relatively effective (Mdn = 4.5; IQR = 4 on a scale from 1 “not effective at all” to 7 “very effective”).

## 4. Discussion

Disasters and pandemics pose unique challenges to health care delivery. The current literature suggests that implementation of telerehabilitation services could provide effective care for children and adolescents, including during extreme physical isolation in the COVID-19 era [25]. However, prior to COVID-19, the literature on telerehabilitation dissemination in pediatric rehabilitation population was sparse [20]. Since telerehabilitation may require tailor-made solutions for specific populations, and in order to develop effective dissemination protocols in the future, our understanding of the factors affecting telerehabilitation provision is crucial. This work detailed the strategy decided upon for transition to telehealth during the initial stages of the COVID-19 pandemic and evaluated this transition by considering both the perspectives of clinicians and families. While our results are specific to an extreme situation, we believe the lessons learned from this experience may inform healthcare practitioners and support future work on pediatric telerehabilitation.

This work describes telerehabilitation principles which were devised by a multidisciplinary rehabilitation team, part of the largest pediatric hospital in Israel which treats children from throughout the country. In the absence of existing guidelines, a solution for telerehabilitation had to be implemented quickly [26,27] but without compromising quality of care. The goals defined by the assembled focus group were based on clinical and professional experience and existing research evidence [28]. Importantly, achieving change in functional status of the children was not one of the a-priori goals, despite the fact that in face-to-face therapy this would a predominant goal [29]. This represents an important difference with respect to non-pandemic conditions. Whether this definition of goals would be sustainable for longer periods of time in telerehabilitation needs to be investigated in future research. It may be also that definition of specific goals, for each sector, would have resulted in better attainment of these goals. Finally, given the conditions, it was impossible to compare goal attainment in telerehabilitation with standard care under the same conditions (since this was not simultaneously provided).

Our results showed that when comparing the different goals, therapists estimated that the ability to maintain the therapeutic alliance was superior to the ability to achieve other goals—and interestingly, this difference was evident also in physical and occupational therapy sessions, both sectors where physical goals, rather than emotional goals, are typically emphasized. These results were in accordance with feedback from parents, who identified the presence of the therapists as very high. These encouraging results address a major challenge for telerehabilitation during physical isolation, which was the need to continue providing personal and professional telerehabilitation care without compromising the “warm” and personal aspect apparent in face-to-face therapies. Indeed, pre-COVID-19 research indicates that healthcare professionals are reluctant to accept telehealth [14,30] since, as some indicate, “the therapeutic alliance can only be established face-to-face”, in spite of research suggesting otherwise [31]. The relatively high perceived sense of presence reported in the current study by caregivers and families may suggest that there is a growing acceptance of telerehabilitation by professionals and clients alike. This may indeed be a result of families’ perception of the current situation—as one parent commented, as “we are thankful for what we have”—but the scores obtained for presence were, in 75% of cases, the highest possible score, and similar across different types of therapy. Future studies are needed to evaluate whether this represents a real paradigm shift with respect to pediatric telerehabilitation implementation [25].

Another finding arising from CETS questionnaire responses was that items related to the child component were scored significantly lower than those related to the session itself or to the parent. The ability to evaluate the status of the child and to efficiently transfer feedback were particularly reduced (Table 2). This may stem from the fact that given the conditions, telerehabilitation was transferred via video and in the absence of additional capacities, e.g., depth cameras [32,33] or other motion capture equipment. This may have limited the ability of therapists, who did not undergo training for use of telerehabilitation, to use the technology optimally. Indeed, existing evidence suggests that assessment in telerehabilitation may be feasible and valid for neurological [34] and orthopaedic [35] conditions (including in children), supporting the idea that training and technology may be key to improving these aspects of telerehabilitation usage [36]. Our results further showed that with younger children, the ability to communicate feedback, grade levels of difficulty and provide guidance for parents was diminished. This suggests that special care should be taken when adapting telerehabilitation treatments to younger children, so as to maximize the potential benefits. In contrast, this work identified environmental characteristics, such as the presence of siblings or the child’s personal toys, as facilitators for therapy. Ideas for use of these facilitators was shared between clinicians treating the same child using the CETS form. These contributed to collaboration among a multidisciplinary team under stressful conditions. Our results further demonstrated that the ability of telerehabilitation to provide coping strategies for parents was relatively high, adding to previous work supporting the idea that telerehabilitation interventions targeting parents and aimed at providing coping strategies may be particularly effective in comparison to those targeting children [20].

Research evidence from different clinical populations, such as people with stroke [6,7], people with heart [8] and musculoskeletal conditions [9], or after surgery [10,11], demonstrates that provision of services via telehealth is at least as effective as face-to-face therapy. The current work, although not directly comparing telehealth with face-to-face treatment, extends previous work to demonstrate that effective telerehabilitation can be obtained across different health professions also under extreme circumstances. This may warrant more attention as protocols for telehealth are developed and disseminated, and the pandemic has accelerated these processes [25,36]. With the new competencies telerehabilitation requires from clinical staff [16], our results showed that clinicians were able (given the necessity) to adapt to the novel conditions. Given that, following the pandemic, the regulatory status of telehealth has changed as well [37], potentially allowing for a change in reimbursement models, it may be that these findings will support a change in the uptake of telerehabilitation services post-COVID-19. Importantly, however, when the lockdown ended virtually all families returned to face-to-face therapy at the department. This suggests that a paradigm shift, were one to take place, may require a longer period of time. In light of these results, developing pediatric telerehabilitation protocols should be prioritized. Now may be the time to create a longer-term solution for pediatric telerehabilitation to maintain continuity of care during such unpredictable times.

Several methodological limitations to this work need to be recognized. First, telerehabilitation sessions were initially suggested to children for whom this line of treatment was feasible (e.g., had internet at home, acceptable cognitive and emotional status). This fact may have generated a bias in the ability to accurately estimate the success of telerehabilitation in the general pediatric rehabilitation. It should be noted, however, that only three children dropped out of treatment due to lack of effectiveness. Another issue associated with generalizability had to do with the fact that telerehabilitation sessions were administered using simple video chats, thus potentially limiting the ability to integrate additional treatment characteristics (e.g., when using motion capture devices etc.). Furthermore, due to time restrictions, and in order not to further burden the families, we did not include family members in our focus group, designing the principles of transition to telerehabilitation. Importantly, the lived-in experience of the families in the process of transition to telerehabilitation should be included in further development of pediatric telerehabilitation care. It should also be noted that, given the unprecedented nature of the pandemic, the ability of the focus group to rely on existing literature in order to build the guidelines was limited, since none of the existing work addressed extreme conditions as faced in the current situation. Thus, the focus group members relied more on clinical experience and group discussions than on existing published principles. This highlights the importance of adding, to existing work, the perspective of telerehabilitation usage during the extreme conditions of a global pandemic. While data presented in this work represent a snapshot of the situation in the early stages of the COVID-19 pandemic in Israel, follow-up investigations of telerehabilitation implementation in pediatric care are warranted.

## 5. Conclusions

In conclusion, the present work describes the steps taken and the responses of clinicians and families to the transition to telerehabilitation in a pediatric rehabilitation setting. We present the guidelines developed for telerehabilitation under physical isolation due to the COVID-19 lockdown, and the views of clinicians and families towards implementation of telerehabilitation services. We demonstrate that despite the need to rapidly deploy telerehabilitation services, high levels of presence were perceived by families during telerehabilitation sessions. Clinicians found the ability to maintain the therapeutic alliance to be higher than other predefined goals. This supports future planning of telehealth deployment and implementation in pediatric rehabilitation in emergency as well as, potentially, non-emergency conditions.

## Figures and Tables

**Table 1:** Guidelines for pediatric telerehabilitation under COVID-19, administered to clinical staff at the department following the work of the focus group.

Topic	Description	Notes to Clinicians
Goals of telerehabilitation	Maintenance of the therapeutic alliance. Provision of coping strategies for parents. Assistance in maintaining routine. Prescribing measures to prevent functional deterioration.	Principles of telerehabilitation under extreme conditions may vary from those of telerehabilitation or standard treatment under usual conditions. Any concerns/questions (ethical, functional or other) you may have can be discussed within the clinical staff in order to facilitate treatment.
Preliminary discussion of therapeutic principles (clinician + parent)	A preliminary talk should include: Expectations of both clinician and parent. Ensuring parental availability during and following session. Checking of physical affordances of the space to ensure safety. Definition of time limits and content of treatment (including breaks).	If parental availability does not match expectations, the use of telerehabilitation needs to be reconsidered. Times of treatment should optimally remain constant between days. Physical space can be checked using video recording by parent. Care should be taken to enable privacy as much as possible (not always possible within lockdown conditions).
Content of session	Duration of sessions should optimally not exceed 30 min. If child is new to the system, the first session is to be introduction. Treatment needs to be divided into short segments (~8–10 min), separated by free play or scheduled breaks, in order to increase cooperation.	The child’s age and cognitive state need to be taken into consideration when determining length of subparts within the session. In the motor domains, simple tasks need to precede complex tasks in order to minimize risk of injury. Gradual increase in difficulty is key. In order to ensure understanding, a “talk-back” approach is warranted, asking child/parent to repeat instructions.
Assessment	During the first meeting, several questionnaires will be filled addressing mental state and needs of families. Following each session, families will fill a short questionnaire. Clinicians will fill a questionnaire following each treatment documenting its characteristics.	If the child was not present at the department pre-COVID, an attempt needs to be made to administer functional assessments.

**Table 2:** Responses from The Clinician Evaluation of Telerehabilitation Service (CETS) (N = 67) and results from PCA. Rotated structure matrix for PCA with Varimax rotation of a three-component questionnaire. In bold are the maximal loadings for each question.

	Score [1,2,3,4,5] Mean ± SD	Rotated Component Coefficients			
Item		Component 1	Component 2	Component 3	Communalities
1. I was able to grade levels of difficulty in the session according to the child’s capabilities	3.38 ± 1.25	**0.939**	−0.026	0.023	0.883
2. Feedback to the child was efficiently transferred	2.92 ± 1.02	**0.911**	0.123	−0.017	0.845
3. I was focused during the session	4.29 ± 0.99	**0.837**	0.040	0.030	0.703
4. Home environment conditions were suitable for the activities	3.29 ± 1.12	**0.791**	0.106	0.009	0.638
5. The child was distracted (inversed score)	2.96 ± 1.43	**0.709**	0.515	0.102	0.778
6. I could evaluate the status of the child	2.82 ± 1.05	**0.680**	0.100	−0.092	0.481
7. The child cooperated during the session	3.79 ± 1.22	**0.623**	0.356	.390	0.666
8. This telerehabilitation session assists the child	3.67 ± 0.96	**0.615**	0.540	−0.044	0.672
9. This telerehabilitation session assists in preventing functional deterioration	3.79 ± 0.83	0.040	**0.916**	0.141	0.860
10. This telerehabilitation session assists in maintaining daily routine	3.54 ± 1.02	0.009	**0.906**	−0.033	0.822
11. This telerehabilitation session assists in maintaining therapeutic alliance	4.17 ± 0.64	0.404	**0.530**	0.272	0.518
12. This telerehabilitation session provides coping strategies to the parent	3.92 ± 0.72	−0.140	−0.029	**0.850**	0.742
13. The parent cooperated during the session	4.71 ± 0.55	0.145	0.506	**0.619**	0.660

**Table 3:** Mean scores of the three-item Therapist Presence Inventory-Client Version (N = 36).

Item	Mean (SD)	95% Confidence Interval (CI) for the Mean
My therapist was fully there in the moment with me.	6.8 (0.38)	6.71–6.96
My therapist’s responses were really in tune with what I was experiencing in the moment.	6.75 (0.6)	6.55–6.95
My therapist seemed distracted (*reversed).	6.67 (1.17)	6.27–7.0

## Data Availability

The data presented in this study are available on request from the corresponding author. The data are not publicly available due to issues of privacy.
